# Supplementary material for: Social and Contextual Influences on Antibiotic Prescribing and Antimicrobial Stewardship: A Qualitative Study with Clinical Commissioning Group and General Practice Professionals
Source: Antibiotics (Basel). 2020 Dec 1;9(12):859. doi: 10.3390/antibiotics9120859 (PMC7759918; doi:10.3390/antibiotics9120859)
Supplement: Supplementary file 1 [file antibiotics-09-00859-s001.pdf]

Social and contextual influences on antibiotic prescribing and antimicrobial stewardship: A qualitative study with Clinical Commissioning Group and general practice professionals

SUPPLEMENTARY MATERIALS

Contents

Supplementary Material 1: Interview topic guides 2

Supplementary Material 2: Additional quotes illustrating the findings 4

1. Immediate context: patient and practice characteristics 4
2. Wider context: pressures on the system and resources 6
3. Collaborative and whole-system approaches 9
4. Relativity of appropriate prescribing 11

Supplementary Material 1: Interview topic guides

Topics explored in the interviews with CCG professionals, with example questions:

- Participants' roles and attitudes to AMS, e.g.:
    - Can you tell me about your role within your CCG and what activities you are involved with in relation to AMS?
    - What other roles in your CCG or team are also connected to AMS?
    - Do you know how antibiotic prescribing in your CCG compared to other CCGs or to the national average?
- Quality Premium (knowledge, communication), e.g.:
    - Can you tell me what you know about the antibiotic Quality Premium?
    - Can you tell me about how your CCG/team were informed about the antibiotic Quality Premium?
    - What advice, if any, did you/team receive in how to meet the criteria outlined in the antibiotic Quality Premium?
- Implementation of QP & communication with GPs, e.g.:
    - What did you/team do about implementing the antibiotic Quality Premium?
    - How did/do you communicate with general practices about the criteria introduced in the antibiotic Quality Premium?
    - How did/do you support general practices in meeting the AQP criteria?
- Implementation of AMS interventions, e.g.:
    - What did the general practices in your area do about optimising AP?
    - May I ask you specifically about [patient facing leaflets / delayed prescriptions] - have you encouraged the GP staff to use these? How?
    - What do you think has gone well / not so well when trying to influence antibiotic prescribing in general practice?
- Overall views on AMS & QP, suggestions for improvements, e.g.:
    - Generally, what do you think motivates general practice staff to change their antibiotic prescribing?
    - Overall, how successful do you think the antibiotic QP was in changing antibiotic prescribing in general practices in your area?
    - How do you think the antibiotic QP could be improved in future?

Topics explored in the interviews with general practice professionals, with example questions:

- Participants' roles and attitudes to AMS, e.g.:
    - Can you tell me about your role in your practice and any activities that you have been involved in to optimise/improve antibiotic prescribing?
    - Do you know how your antibiotic prescribing in your practice compares to other practices in your area or to the national average?
    - How important is it for you and your practice to optimise antibiotic prescribing?
- Quality Premium (knowledge, communication), e.g.:
    - Can you tell me what you know, if anything, about something called Quality Premium?
    - How familiar are you with indicators or targets related to antibiotic prescribing?
    - Do you know about any financial incentive schemes in your area and what targets they include?
- Communication between CCG and general practice, e.g.:

    - How does your CCG team communicate with your practice about (optimising your) antibiotic prescribing?
    - What has your CCG asked or encouraged your practice to do to optimise your antibiotic prescribing?
- AMS interventions, e.g.:
    - Can you tell me about any strategies that your practice has implemented to optimise your AP?
    - Can you tell me more about [patient facing leaflets / delayed prescriptions] – have you used them? How?
    - What has worked well / not so well in your practice in terms of improving antibiotic prescribing?
    - In general, how does your practice team implement new interventions to change practice?
    - What is most difficult when trying to introduce change in your practice team?
- Overall views on AMS & suggestions, e.g.:
    - What do you think would be most helpful for your practice in optimising antibiotic prescribing?
    - What resources or support from your CCG would your practice need to help optimise antibiotic prescribing?

Supplementary Material 2: Additional quotes illustrating the findings

1. Immediate context: patient and practice characteristics

Patient population characteristics

The key disease areas [in the borough] include COPD, a lot of infections and again the demography that then reflects on social circumstances and education had over the years created a demand with general practice for doctors who have managed that demand by prescribing antibiotics because a good proportion of our population would see the GP as the first port of call if they are unwell, and this is relative to other areas, the areas with better demography, better economics and better health, better education and more likely to self-care, so even the simple over-the-counter medications, from treating coughs and colds, living with a sore throat etc. Over 50 years there's been a culture that you'd go to the doctor… [and] you might expect to get antibiotics if you have a sore throat. […] A practice at one end uses twice as many antibiotics per one thousand patients than the practice at the other end, but the practice at the end that is using less is often in a better area within the borough, and that will back up my earlier claim that demography, education and being able to afford to self-care. That's not exclusive but that is the variation… the patients who are in the not-so-good areas tend to have greater ill-health, more likely to smoke, COPD and so on. [CCG-13, high-prescribing]

[General practice] requires a whole system rethink as to increase in methods on self-care and that's harder to achieve in some areas where there's perhaps more socioeconomic problems and people go to the doctors more readily. Again, that's quite a simplification of a very complex area but I think where you've got greater health needs associated and greater social needs and social deprivation, then antimicrobial usage is harder to challenge in those areas in my experience. [CCG-9, multiple CCGs]

They tend be areas where there is deprivation or where the patients have high expectations so they're worried and they also get that demand, they want – I want antibiotics. I had it last time, it helped and then the GPs sometimes find that quite hard to say no but then you also get the ones where – what do they call it? The mother in law syndrome where they all live in one household and then the child's unwell, they expect that patient to come back with antibiotics, the mum to come back with antibiotics so cultural issues I would say. [CCG-7, medium-prescribing]

We've got a really complex paediatric population and I think for those patients... we've got a number of patients that are prescribed long term prophylactic antibiotics by secondary and tertiary care and that drives up consumption. And the other one is, we've got a lot of young people, families with young children. They might be living in poverty or in depravation and some of them might not have the educational ability or skills and the lowest socio-economic group and actually they might be coming into practices to demand the antibiotics and they just kind of pop the prescription and the GPs are risk-averse and they are prescribing the antibiotics to the children… [CCG-16, high-prescribing]

Our GPs struggle to say 'no' if I'm honest. We do have, I would say, a reasonably affluent population who are quite demanding, so I don't know whether there's an element of patient demand. I also know that a lot of our practices are quite small, and I don't know whether there are pressures on time in terms of having that discussion with patients about why an antibiotic is or is not appropriate. [CCG-2, medium-prescribing]

I don't think the range of rates have changed significantly but we serve a very low economic group with high levels of deprivation and comorbidity so I think relative to our population we do prescribe

antibiotics, especially COPD our rates have increased so and that's an average spec size change in prescribing [unclear] - possibly prescribing a bit more for those group of patients. [GP-14, medium/high-prescribing]

Practice and prescriber characteristics

We've had quite a few practices that have really had problems with their staffing, with having lots of locums in, being utterly deluged with needing to see patients and appointments etc., and just being overwhelmed with workload and not really having the time to focus any attention and thoughts on what antibiotics should we be using… [CCG-2, medium-prescribing]

There's also something about locums. One of the reasons that we are given from our higher prescribers is that it's the locums that prescribe so I don't know whether they prescribe antibiotics less considerably so whether there is a lack of ownership when you know you're not going back to that practice… [CCG-2, medium-prescribing]

It might be helped by the fact that we're quite small, so we don't have large numbers of patients which I think makes it easier. We can have more continuity of care, doctors can see the same patient, they can know them well. [GP-2, low-prescribing]

…we've got locum doctors here. I think they were prescribing more in comparison to other lead doctors. So then obviously you know their relationship or commitment towards the practice sort of obviously was one reason to prescribe you know more, but obviously we [inaudible] we sort of gave them all the information, you know guidance so that helped eventually. [GP-13, medium-prescribing]

A lot of it I think goes down to some individual GPs in a practice. (…) I have had some GPs say to me that they all follow the rules and they could identify who in their practice is doing all the inappropriate prescribing. Sometimes it's meant that a GP in a practice have had to go with the data and go and have a chat with that individual GP to kind of educate them and say, look, this is all the prescribing that's going on and it's all you that's doing it and just confront them with the data etc., and that seems to work quite well. When they've done audits within a practice and they've been able to show the data to show the difference in prescribing from each individual prescriber in the practice, they seem to think that has worked quite well with some practice to sort of rogues who are doing this inappropriate prescribing. [CCG-3, low-prescribing]

It tends to be pockets of individual practitioners particularly. Some of the older ones who don't always understand the issues or have always, for example, used Co-amoxiclav, even though you're trying to tell them that they shouldn't be using it now first-line, so it's about targeting individuals largely. [CCG-11, low-prescribing]

I think it is just historical and what they're used to and what they're comfortable with and it's what they've always prescribed. […] The practice in question I did go twice to go and do training and education and I did email to and fro with their practice manager about it, but I think that the habits just didn't seem to change. […] I think there are just some GPs that just don't want to change. There always are people that don't like change, that want to just continue as they are. [CCG-3, low-prescribing]

I suppose the problem is the sepsis agenda is now there and people were missing sepsis so it's now about making people comfortable that they're not going to miss sepsis or are giving strategies for delayed prescribing while staying safe in terms of patient care. It's complex and there's two conflicting issues here, antimicrobial resistance and not missing sepsis, and that's what practices are

struggling with at the moment so we're trying to help them with that by having this sepsis lead and myself and the sepsis lead talk about this, just about having this whole system approach to aid diagnosis and rational prescribing with antibiotics. [CCG-5, high-prescribing]

When we look at the results, we can say this prescriber, in this practice appears to be quite distinctly different from all their colleagues within that practice. So my discussions with them at the time were along the lines of: you as a practice have to draw your conclusions from this because only you will know it's whether because that particular prescriber A needs upskilling, B runs the kind of clinics where they see more people with infections than you do, or you're all part-time and that person works full-time. What are the factors that are influencing why their figures are quite distinctly different from the rest? [CCG-19, medium-prescribing]

I think one of [GPs] in particular has a tendency to – well, I know his feelings about antibiotics. I know that whenever we discuss the fact that we're high prescribers, he always feels that we're prescribing for a reason, and that if you were able to compare the data between hospital admissions and prescribing that we would find that our high prescribing actually has a benefit in that we help patients to avoid serious infections. [GP-4, high-prescribing]

2. Wider context: pressures on the system and resources

Pressures on the system and resources

An indicator just by itself is not helpful without resource and I think that's where we're really struggling now. We've got a much more reduced team than we had a couple of years ago and we just haven't got the capacity to be able to do a lot of work on it, so that's where we're at. We're coming up with other solutions but it's got to be within the capacity that we've got at the moment. [CCG-9, multiple CCGs]

[AMS is] probably I'd say less than 5% of my time. It's one of those areas where it doesn't really sit within my role. […] I don't feel very supported basically. I feel that the enthusiasm and time that I put into it is because I think it's an important area, not because I have the time to do it or necessarily want to do it but I know that if I don't do it then no one else is going to pick the workload up so I feel that I do it because it's not going to get done otherwise… I end up doing is just doing it in little bits and pieces and not having enough time to do it properly. There's lots of ideas and things that I would like to do but I don't have the amount of time to be able to do it. [CCG-3, low-prescribing]

I've always been pushing for a dedicated technician that would enable me to collect data, to collect whatever it is I require… if we had a technical support to be able to do the work that we do, both across the interface, I think that would be really helpful. At the moment we're running around like headless chickens just trying to do everything. It's hard work but I think we're motivated and I love what I do and that's what keeps me going. [CCG-17, medium-prescribing]

…the actual workload, I suppose, for medicine management teams, and certainly for myself, just seems to be ever increasing. It's not in relation to antimicrobials. The more it increases elsewhere, the less impact I can have because more of my time is taken away. [CCG-19, medium-prescribing]

The reality is it's all about competing interests. Most people are working 12 hours days. They're working non-stop. They're working very hard. What do you say to them? You can't just keep saying to them, just do more. By more, I don't mean prescribe more but work to try and look at what you're doing. We've got to be looking at ways to incentivise, to take workloads down. To get more people employed in the primary care. There are so many things going on. That's the problem, the context of this research is what's in the background of what's going on in the NHS at the moment. We can't

divorce ourselves. If you ran the same questions probably in 2010, the options of what's available to you would be very different. [GP-06, low-prescribing]

[Auditing antibiotic prescribing is] a much larger piece of the work and so the [CCG's] Medicines Management group doesn't have time to do that. If they don't have time to do that, they're hoping you're going to do that instead. The problem is I could do millions of things. It's about competing: am I going to do this thing or am I going to do something else. It's not actually am I going to do this thing or am I going to have a round of golf. [GP-06, low-prescribing]

The reality is you're talking about a system that is massively overworked. Significantly reduced funding over the years with a massive increase of workload… You're taking systems that are massively under pressure and you're then trying to say, we'd like you all to be perfect. And if you take systems that are trying to survive, stay open, the staff and look after the patients, the initiatives that you're talking about are obviously going to be much lower down that list. [GP-06, low-prescribing]

I just think that some practices just haven't physically had the time to think about it properly and do it. It's just not an urgent priority for them. I think that's been the problem in some practices. [CCG-3, low-prescribing]

We are swamped by the demand from patients in terms of the demand for access so we'll have so many patients to see and it's difficult to sit back and say, right we need to look into this, make changes. You need somebody to study the situation, come up with a solution. Introduce or implement the solution and be available to trouble shoot as necessary. I have been able to do it because I'm part time. I only do two or three sessions a week, having retired, or taken my pension. I actually spend more time than that in doing this because I'm quite interested in the issues. But other practices may not have that facility and they may have difficulty in introducing the change. [GP-07, high-prescribing]

Unfortunately, everyone is very busy and the NHS is under a lot of pressure at the moment. […] I think practice feel under siege and I think that practices need to be engaged, they need to feel that they're doing this for a reason. [CCG-5, high-prescribing]

People have quite limited resources in terms of time, and CCGs have limited resources in terms of money, and there are an awful lot of competing priorities… it is just a really difficult time at the moment in NHS, financially, and I think people have a lot of competing priorities. [CCG-8, low-prescribing]

AMS initiatives need to fit within the constraints and be tailored to local context

…anything to try to help, try and reduce prescribing in primary care, can't increase the workload… there's no point in designing a strategy to reduce antimicrobials in primary care that involves more work because that's unlikely to be taken on board whereas the things that have been done so far, such as the delayed prescribing, patient leaflets, patient information, are efficient, they're very time efficient and they're much more likely to be taken up. [GP-01, low-prescribing]

…my main barrier is finding the time to concentrate on it… I suppose if there was enough of a financial incentive to allow me – for us to get locum cover so I can take a day out of my usual GP work for me to do this audit and just to work it through, that would be a help probably. [GP-4, high-prescribing]

Something can be done by just sending over the report to practice and leave it at that. The thing is that we have been doing that to practices for decades, every three months we give them a prescribing report, they look at it and through it in a bin. Not everybody does it, some people are looking into it and tried to change practices but many will struggle a lot, there are a lot of [inaudible] users. The thing that is most important factor is that it needs to be looked at so I think that they need to approach practices that are struggling with their stewardship or prescribing generally. Antibiotic stewardship or prescribing it would be one little part of the inappropriate prescribing so that would be generalised issue with the rest of their prescribing as well. [GP-15, low-prescribing]

…if you want me to do something, from a prescribing perspective, if it's going to take me time to do, over and above what I normally do, why would I want to do it? If you're going to ask me to do something that's going to save me time, or lessen my burden of workload, then I'll be very interested. But if it's going to increase my burden of workload, I'm not going to be very interested at all. I guess it's trying to think of ways in which we can ask general practice to take things on in ways they may not necessarily obviously think of. [CCG-19, medium-prescribing]

…if an educated person who's a GP doesn't implement a policy that you think is sensible, you need to go and really listen to the reasons why they are having difficulty with implementing. Then you need to think about whether those reasons are reasonable, and then you need to think about how you can counter those reasons. [CCG-8, low-prescribing]

I think we've got various different models of how practices operate in terms of whether they have a CCG pharmacist working in them, whether they employ their own pharmacist, whether they don't have any but they might have an interested GP who provides some capacity to do audit. There's no single model across all of the CCGs so it's about trying to tailor the response to the individual practice really. [CCG-9, multiple CCGs]

I think it's almost like an organisation's development issue as opposed to just a clinical issue. You might be telling them something and they might fundamentally know what you're telling them is reasonable but if they haven't got the means to make this stage happen for whatever reason and because the practice is in a bit of a mess, then there's no point in keeping on telling them 'Oh you need to change your prescribing. This isn't right. That's not right.' You've got to sit back and say 'Right, how can we help you and what changes may be needed to be made to change the infrastructure of your practice to make that happen?' That's far more challenging and far harder to make that happen for all sorts of reasons. Lack of capacity and people's lack of honesty about where there are general problems that need to be addressed. [CCG-9, multiple CCGs]

I will usually talk to the prescribing advisor first who [inaudible] leads the practice to have a little look on the system and just do a really snapshot audit and just see if anything unusual had happened. And then if we think something has changed - it can sometimes be they might have a new prescriber or something - then we'll go out and talk to the practice about it; or we might just send them the audit and say, 'Here you are, and here's some recommendations, let us know if you want us to come and talk to you about it any further'. It does depend on the practice as to how they will let us in as such, but we've got a good relationship with most practices to be able to do that. […] The prescribing advisors will go out and help them if they need to. If they do want an audit done, then we will do that. If they want to talk to them, then we'll do that too. We point them in the right direction for the different resources that are available. [CCG-1, low-prescribing]

3. Collaborative and whole-system approaches

Communication, teamwork and learning from each other

The guys that I work with are really pretty good. We discuss things between us as well. We're not like a closed door. I'm the senior there, but everyone comes in and discusses things with me and we'll sit down and we'll chat about them and they'll go, "What do you think? You know, we've had this. Where shall we go from here? What shall we do?", and I think that's what you need. Not to be like a closed door where you sit in your little room and you just give out whatever people ask you for. I think it's important to discuss things and then to all say the same. [GP-11, high-prescribing]

I think it may be just getting us all together may be slightly difficult. I think once we were all together and agreed I don't think it would be necessarily too difficult. We're a small team. I think it's having the time and the continuity and the communication and I think if we were all together on something and agreed this is what we need to do and this is how we're going to do it I don't think that would be too difficult to implement. As I say, because we are quite a small team, it's drawing that team together and getting us all to sing off the same song sheet. Time would probably be the biggest challenge. [GP-17, medium/high-prescribing]

I work quite closely with our counterparts, so I have very close working relationships with microbiologists and our clinical lead in [county] healthcare trust, so we work quite closely with our hospital foundation trust in terms of joint policy, trying to work together so that we're tackling issues across the whole interface so it's not just in primary care, but looking at the issues between secondary and primary care as well. So we try and produce joint guidance wherever possible. [CCG-14, medium-prescribing]

I suppose experts from areas where it's gone well, I'm happy to take advice on what I can do better. I'm sure I can do a lot more, but I do feel I'm working a bit in isolation even though I do get to meet other colleagues sometimes. [CCG-6, high-prescribing]

On a one-to-one basis our prescribing support pharmacists work with the practices to highlight where there's deviation, I guess that's the key thing. It's about personal relationships really and a good working relationship. [CCG-22, low-prescribing]

I made the contacts and made the relationships with the practices… They do take a lot of it on board and you can see that they are quite well engaged and I get a lot of queries coming through. Anything that seems very strange to them, I'll get GPs emailing me. So I can see that, you know, it's not been overnight, it's taken many years to get to the place where I am and to build the relations. But what I feel is a really important part of the job, to build the relations, to be respected in what you do and people value your opinion. [CCG-17, medium-prescribing]

Leaders, champions and relevant experts

The other thing is what changed in the last couple of years, the leadership around antimicrobial stewardship in NHS England. I can't tell you how helpful [name] has been. We had not had that kind of access before. She personally shares a lot of stuff, creates groups, attends primary care forums. The leadership in antimicrobial stewardship under [name] has been fantastic. [CCG-10, medium-prescribing]

We also had one of the microbiologists from the hospital who came and went through the results so that if the GPs had any questions that he was the right person there to be answering the questions. I

think that worked quite well, having a specialist there that they could ask questions to… [CCG-12, high-prescribing]

I've had one experience of one large practice where the GP prescribing lead was very, very engaged and he did manage to get a lot of his other GPs to take a stance on the antimicrobial stewardship and really start to examine their prescribing. [CCG-6, high-prescribing]

More than anything I think it's probably about leadership within the practices themselves. I think those that feel very strongly about it and have a very coherent plan about all aspects of prescribing tend to be the ones that have a coherent plan with regard to antibiotic prescribing. What I find is that antibiotic prescribing tends to be almost like a surrogate marker for good and bad prescribing in all aspects of prescribing. [CCG-4, high-prescribing]

I think the rate of success is where, and I mentioned each practice has a nominated antibiotic guardian. It's more successful when they take a lead on an ongoing basis to maybe scrutinise antibiotic prescriptions and feedback. I described to you the CCG , practice pharmacies would discuss it twice a year in these meetings and that may be the only time, but where a practice took the reins really and decided it was something they wanted to do and they did implement something and all agreed to do it, was, 'Right, we're going to use deferred or delayed prescriptions,' so where a practice is cohesive and you have that leadership, then you're more likely to get sustainable change , and it's also influenced by the regular GPs in the practice so where there's not a turnover of GPs, where there's not regular or irregular locum GPs coming into the practice. I can't be absolutely – they will be all factors that have some influence on a successful practice compared to the one that's not been successful. [CCG-13, high-prescribing]

Somebody with the knowledge and the authority that I have in my practice would be useful [for practices]. Again, I have partners who would be happy to take up my suggestions, whereas I know of colleagues where partners do not interact and partners do not help each other or accept each other's suggestion. [GP-7, high-prescribing]

…having an antibiotic champion if you like, an antibiotic steward champion, stewardship champion. I think that's a good idea. We don't have that but actually that's a really good idea. You've got a lead for a lot of other things, whether that be cancer or palliative care, safeguarding… you've got leads for a lot of high-risk conditions, vulnerable adults, vulnerable children. You've got leads for all these and actually this is another high risk condition potentially that you could have a practice lead for this, takes an overview to make sure and just remind people and looks at prescribing or looks at conditions or takes on an initiative every so often. So that might be a way to keep the awareness. 'Cause as I said we get the awareness once a year maybe and it's in all the journals, so everybody is aware of it but what we need to do to get it high up on the practice priorities. It happens sort of once a year and the way to keep it up there might be to have some kind of practice lead. [GP-01, low-prescribing]

Consistent and whole-system approaches

Sometimes the feedback we get is 'if I don't prescribe [antibiotic], then [patients] will just go to out-of-hours and get one', and I think that is an issue even though we've done some work with out-of-hours… we found that if a practice can be united in their approach to managing antimicrobial prescribing, then that seems to reflect a kind of lower overall antibiotic prescribing rate and that's true of a lot of prescribing indicators. If you've got people working as a practice and not as a set of individuals, that's really really helpful. [CCG-9, multiple CCGs]

I think what they've got is a very coherent plan as an entire practice. Sometimes what you can see, you can see it on the journal entries for individual patients, sometimes they keep coming back until they find the doctors that tend to be the high prescribers – 'they' being the individual patients. So they almost re-consult. So you might have one or two good prescribers within a practice but unless there's the same approach adopted by all prescribers, then it seems to fall a bit flat if patients seem to recognise there's a way around that system. It's about patient education as well. If patients themselves can understand that they've got a self-limiting condition that doesn't need antibiotics that will help reduce consultation rates for self-limiting condition but if they're encouraged by one or two prescribers that antibiotics will help them get better, then they won't learn, and it doesn't really change anything over time. [CCG-4-4Q]

We deliver events around antimicrobial resistance to practices normally at a neighbourhood or locality level so not to individual practices but to groups of practices. We also liaise with the Trust and we forge links with the consultant microbiologists to sort of further the antimicrobial stewardship agenda. And also the Community Trust; we link into the [area] Community Health Trust and their antimicrobial pharmacists and stewards and we've tried to have a consistent approach across the whole city to reducing antimicrobial prescribing. [CCG-5, high-prescribing]

When we did some work with community pharmacists, we also gave them the resources as well and showed them the resource that we were supporting our GPs with so that they were kind of singing from the same hymn sheet, so they were also aware because I'm not sure that community pharmacy get the education that they necessarily need for, or they don't always know what's happening in primary care so we kind of try joined… the message joined up this year or for the last few years. [CCG-7, medium prescribing]

I think it could be joined up with other bits, so like it could join up the secondary care bit or the campaign side of it. It needs to be a kind of a whole system plan. I don't think it helps that it seems to be too bitty, so I think... I would say it needs to be a bit broader but no deeper. [CCG-16, high-prescribing]

4. Relativity of appropriate prescribing

Relativity of 'appropriate' prescribing

Sometimes the targets can feel quite arbitrary, it is difficult because it's hard to get a target that suits everybody… […] Interestingly the Trimethoprim part of it so reducing the Trimethoprim, I felt that the microbiologists weren't massively impressed with that which was interesting. They've kind of gone with it but I think… I think they didn't think it was going to make that much of a difference so that was interesting. [CCG-1, low-prescribing]

I would imagine the E. coli indicator is less well met because it's very difficult, because it's not clear exactly what you need to do to meet the E. coli objective. Clearly, we all know about the UTI in care homes work so that's an aspect, but E. coli isn't exclusively UTI. There are lots of other conditions, wound care, prostates, all sorts of things; it's much more difficult because it's not so clear-cut. [CCG-8, low-prescribing]

It almost seems like people just pluck these things out of thin air and it's who? Do they even speak to people in the field and doing this job on a daily basis? When we did provide some sort of feedback on the QP, we just got nothing back. I still haven't had my question answered about biliary sepsis and biliary sources of infection. I'm still waiting. It's been over a year now. Nobody's answered the

question. Actually, on the contrary, I was told, if you think of something else, can you let us know. [CCG-17, medium-prescribing]

I think the thing that's a bit more controversial at the moment is the Trimethoprim and Nitrofurantoin in the sense that I think there's some feeling that evidence base for that was a bit rushed in terms of some assumptions made that actually switching to Nitrofurantoin is the right thing to do. I know there's been some sort of reluctance from some microbiologists in saying actually if Trimethoprim resistance is low a specimen is [inaudible], then you should still use it. […] What are actually trying to achieve and why are we trying to achieve it and is it reasonable? So I think some of the thinking on that is being done now as opposed to: why did we introduce a target on it if we haven't done the thinking? It's just a bit rushed but as I say, that's just the sort of things I've heard, and I think the jury is still out on that really. [CCG-9, multiple CCGs]

…as an average across the CCG we want to achieve the national requirements. We want some practices to be under, and some practices will be over. [CCG-8, low-prescribing]

Nationally [antibiotic prescribing] compares very well if you look at it at [county] level but obviously we don't aggregate it at [county] level anymore because there is no [county-level] singular organisation. So when you look at it across the [multiple] CCGs, there's quite a variation. Historically [one CCG] has had very good, very low prescribing rates of antimicrobials. (…) but [another CCG] has remained a bit of an outlier… Different demographics, different ethos to a lot of general practice in [the other CCG]. I'd say generally speaking [county] prescribing is compared very favourably nationally but with the exception of [CCG]. [CCG-9, multiple CCGs]

In relation to the CCG, I think we're on the lower end… We're in what they call their green category. [GP-5, high-prescribing]

I'm not sure how accurate [comparing prescribing data] is and the reason being is that I look at COPD patients… I tend to prescribe a lot more for that specific indication. One of our female GPs who deals with women's health does things like Azithromycin and antibiotics specifically more for female gynaecology problems, she may have higher rate of prescribing for that particular area. [GP-14, medium/high-prescribing]

We've been criticised in the past for having a higher, it was either Co-amox to Amoxicillin or Quinolone to Beta-lactam, the ratio and we wondered whether that was because we are a relatively low-prescribing practice and so we tend not to give out Beta-lactams without meeting the guidance, which means we would target the Quinolone for conditions such as prostatitis, or where there was a genuine Beta-lactam allergy or in a urinary tract infection or a pyelonephritis. So we wonder whether it's because we have more specific prescribing that we've got a higher ratio. [GP-01, low-prescribing]

The only one that I have as a high prescriber is the walk-in centre and the only reason that one comes up as high prescribing, as in red, is because actually the STAR-PU value is wrong. They have a registered population but they also see any walk-ins as well. I think their registered population is about 3,500 but they don't have two codes. What they really need to have is two codes. One code for the walk-in element and one code for the registered population. Then the problem is that we can't monitor the walk-in element, because in order to have the STAR-PU, you need to have a registered population. That's why the data is skewed for them. That is my only red practice. The rest of them are all either the lower end of amber or green. [CCG-17, medium-prescribing]

The only thing which we are a bit higher on is the antibiotic prescribing for Augmentin and Cephalosporins. We have had a look at all the patients who were prescribed this. I think they're all complicated patients who are under the [area] children's hospital and [inaudible] so there has been

a valid reason why they've been on these prescriptions. I think it's following the audit. [GP-09, medium/low-prescribing]

You've got to understand, the prescribing is not just about the prescriber, it's also about understanding the prescribing environment that they're working in, if that makes sense. It probably doesn't take a genius to work out, that the one that was the highest was also the one doing the most emergency work. Because if you've got 50 people sitting outside your room, metaphorically [unclear] for blood, probably spending, you know, an extra 10-15 minutes of your consultation about why no antibiotics maybe more challenging. [GP-06, low-prescribing]

Changing social norms about appropriate use of antibiotics

I think the majority of practices are concerned about this. They're all clinicians, they all understand the consequences of antimicrobial resistance and that if we don't do something about it in the next 10-15 years, then this will have consequences for their children and their children's children… [CCG-5, high-prescribing]

I think attitudes are changing and we are making progress with regard to not having to prescribe for clearly viral infections or situations where there are symptoms but the symptoms do not suggest an active bacterial infection. [GP-07, high-prescribing]

I think we have the old population, it's certainly where there's a high expectation for antibiotics but I must say that the work that's been done from Public Health and all the work that's been done on the BBC to raise awareness about antibiotic prescribing I find that patients are asking less, or demanding less. [GP-16, medium-prescribing]
